# L-Carnitine and Male Fertility: Is Supplementation Beneficial?

**DOI:** 10.3390/jcm12185796

**Published:** 2023-09-06

**Authors:** Filipa G. Mateus, Silvia Moreira, Ana D. Martins, Pedro F. Oliveira, Marco G. Alves, Maria de Lourdes Pereira

**Affiliations:** 1 Department of Medical Science, University of Aveiro, 3810-193 Aveiro, Portugal; mateus@gmail.com (F.G.M.); s.moreira@ua.pt (S.M.); 2 LAQV-REQUIMTE and Department of Chemistry, University of Aveiro, 3810-193 Aveiro, Portugal; anacdmartins@gmail.com (A.D.M.); p.foliveira@ua.pt (P.F.O.); 3 CICECO-Aveiro Institute of Materials, University of Aveiro, 3810-193 Aveiro, Portugal; 4 iBiMED-Institute of Biomedicine, Department of Medical Science, University of Aveiro, 3810-193 Aveiro, Portugal

**Keywords:** L-Carnitine, dietary supplement, male infertility, Sertoli cells, reactive oxygen species, antioxidant, sperm quality, reproductive health

## Abstract

L-Carnitine, a natural antioxidant found in mammals, plays a crucial role in the transport of long-chain fatty acids across the inner mitochondrial membrane. It is used as a nutritional supplement by professional athletes, improving performance and post-exercise recovery. Additionally, its therapeutic applications, including those in male infertility, have been investigated, as it may act as a defense mechanism against the excessive production of reactive oxygen species (ROS) in the testis, a process that can lead to sperm damage. This effect is achieved by enhancing the expression and activity of enzymes with antioxidant properties. Nevertheless, the mechanisms underlying the benefits of L-Carnitine remain unknown. This review aims to consolidate the current knowledge about the potential benefits of L-Carnitine and its role in male (in)fertility. Considering in vitro studies with Sertoli cells, pre-clinical studies, and investigations involving infertile men, a comprehensive understanding of the effects of L-Carnitine has been established. In vitro studies suggest that L-Carnitine has a direct influence on somatic Sertoli cells, improving the development of germ cells. Overall, evidence supports that L-Carnitine can positively impact male fertility, even at a relatively low dose of 2 g/day. This supplementation enhances sperm parameters, regulates hormone levels, reduces ROS levels, and subsequently improves fertility rates. However, further research is needed to elucidate the underlying mechanisms and establish optimal doses. In conclusion, the role of L-Carnitine in the field of male reproductive health is highlighted, with the potential to improve sperm quality and fertility.

## 1. Introduction

In recent decades, infertility has emerged as a significant challenge for numerous couples worldwide. According to the World Health Organization (WHO), infertility is defined as the inability of a sexually active couple without contraceptive methods to achieve pregnancy within one year. Infertility is now recognized as a medical condition, and recent statistics indicate that 50–80 million individuals are affected by this this problem, with 20–30% of cases attributed to male factors [1]. Considerable efforts have been made to diagnose and understand these cases. The diagnosis of male infertility relies primarily on semen analysis, which involves the evaluation of sperm concentration, morphology, and motility [2]. Currently, modern male fertility treatments involve medication that stimulates the release of hormones that enhance sperm production, like clomiphene citrate [3], and in more severe cases, assisted reproductive techniques (ART) like in vitro fertilization (IVF) alongside surgical interventions, such as varicocelectomy and vasectomy reversal. While effective, these methods remain out of reach for certain couples and may bring about complications for female partners, including ovarian hyperstimulation syndrome. It is important to explore more treatments to address male infertility in order to reduce side effects and to be assessed by more people [4]. This analysis provides essential insights about potential causes and helps guide appropriate treatment options. However, it is important to note that infertility is a complex problem with various contributing factors. While semen analysis is a valuable tool for assessing male fertility, it does not capture the full spectrum of possible causes. In some cases, additional investigations such as hormonal profiling, genetic testing, or imaging studies may be necessary to identify underlying conditions or abnormalities that affect fertility. Furthermore, the problem may be upstream, during spermatogenesis, responsible for the production and maturation of sperm cells. 

In mammals, spermatogenesis is primarily regulated by Sertoli cells which play an essential role in supporting and nurturing the survival, proliferation, and differentiation of germ cells [5]. It involves three key stages: proliferation and mitotic differentiation of spermatogonia, the meiotic phase, and spermiogenesis [6,7]. During the initial phase, spermatogonia undergo a phase of proliferation and mitotic differentiation, originating primary spermatocytes, precursors to fully developed sperm cells. The subsequent stage is known as meiosis and involves specialized cell division of primary spermatocytes, resulting in the formation of haploid spermatids. In the final stage, spermatids undergo morpho-functional changes, including the development of a tail (flagellum) and the formation of a compact head containing genetic material, and the acrosomal vesicle, important for fertilization [8]. Mature spermatids are released into the seminiferous tubules, where they become spermatozoa capable of fertilizing an egg [5]. At last, in the mature stage, spermatozoa are released by ejaculation, where they gain motility so that they can go toward the oocyte and trigger fertilization [9].

There are many male factors that contribute to pathologies in the male reproductive system. A well-known example is the overproduction of reactive oxygen species (ROS), responsible for up to 30–80% of structural damage in sperm [10]. From a clinical perspective, studies have reported that infertile men tend to have higher levels of ROS with a detrimental impact on sperm quality and pregnancy rates [11]. Certain levels of ROS are required for the maturation of spermatozoa, acrosome reactions, capacitation, hyperactivation, and sperm–oocyte fusion. However, when the levels of ROS overcome total antioxidant capacity, there may be a negative impact on sperm parameters [12]. Poor semen quality has been identified as one of the most significant factors affecting reproductive health [13]. ROS are unstable oxygen-containing molecules that easily react with other molecules in a cell [14] and are generated after physiological dysfunctions or exposure to physical agents, such as ultraviolet rays and heat, chemical factors, environmental toxicants, smoking, alcohol, or even other factors, such as obesity, varicocele, aging, or diabetes [11]. ROS are usually produced by spermatozoa via the mitochondrial electron chain [15]. At higher levels, when ROS levels overtake cellular antioxidant defenses, it leads to significant DNA damage, increased lipid peroxidation (LPO), and oxidative stress (OS). This detrimental process compromises mitochondrial functionality, subsequently affecting the capacitation of sperm cells [12]. Additionally, it can affect the hypothalamic axes, disrupting the secretion of sex hormones and then sperm parameters [16]. ROS can trigger OS, which negatively impacts sperm motility, morphology, and DNA integrity, resulting in reduced fertility [17]. Additionally, elevated LPO can adversely affect the functional integrity and fluidity of the sperm plasma membrane, which contains high levels of polyunsaturated fatty acids (PUFAS) [18]. This process leads to the generation of malondialdehyde (MDA) and 4-hydroxynonenal (4-HNE), further compromising sperm quality [19]. 

Antioxidants have been reported as part of possible therapeutic strategies to reduce the imbalance between the natural antioxidant defenses of the body and ROS, thereby improving fertility [11]. They can be classified as enzymatic, converting dangerous oxidative products into hydrogen peroxide (H_2_O_2_) and then into water, like superoxide dismutases (SODs); or non-enzymatic, being produced or consumed endogenously from food or supplements, thus interrupting the chain reactions of free radicals [20]. Among the latter, we can find publications on L-Carnitine in the literature [21]. Antioxidants are naturally found in the semen, although it is reported that the antioxidant capacity of semen is reduced in infertile men when compared to fertile men, contributing to the imbalance between antioxidants and high levels of ROS [22]. L-Carnitine is described as a powerful antioxidant that reduces OS in several tissues, including the testis and epididymis. It acts by scavenging ROS and preventing their damaging effects [23]. Indeed, L-Carnitine (β-hydroxy-γ-trimethylaminobutyrate), a quaternary amine, is known as a natural antioxidant found in mammals. Overall, 25% of the daily L-Carnitine required is produced endogenously, synthesized in the liver and kidneys from amino acids such as lysine and methionine, while the remaining 75% is provided by food intake such as meat, milk, and fish. L-Carnitine is primarily recognized for its role in facilitating the transport of long-chain fatty acids across the inner mitochondrial membrane [24]. This process enables the efficient utilization of fatty acids as an energy source within mitochondria. By aiding in the transport of fatty acids, L-Carnitine plays a crucial role in supporting cellular energy production and metabolism [24]. In humans, the daily synthesis of L-Carnitine by adults normally ranges from 11 to 34 mg, equivalent to approximately 160–480 μg/kg of body weight [25]. However, these synthesis rates can be influenced by several factors, such as stress, specific diets like vegetarianism, or physical exercise, which can result in L-Carnitine deficiency in the body [26]. L-Carnitine deficiency can cause muscle weakness, metabolic disorders, cardiovascular issues, and in severe cases, neurological symptoms [25]. In both men and women, L-Carnitine is predominantly concentrated in skeletal muscle, with levels ranging from 50 to 200 times higher than those found in the blood [26], on average, with concentrations between 25–50 μM for a healthy adult [27]. Notably, the highest levels of L-Carnitine are found in the epididymis, where concentrations can be up to 2000 times higher than those observed in the blood [28]. Furthermore, there exists a positive correlation between the content of free L-Carnitine in seminal fluid and sperm count and motility [29]. In addition to this, research has established that infertile men commonly exhibit diminished levels of L-Carnitine in their seminal fluid [30,31,32]. These findings highlight the critical role of L-Carnitine in maintaining optimal sperm quality.

**Figure 1:**
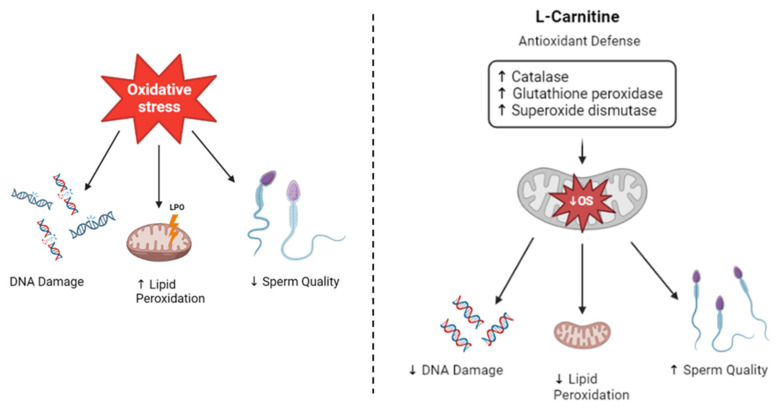
Antioxidant properties of L-Carnitine. Oxidative Stress induced by reactive oxygen species (ROS) leads to DNA damage, increases lipid peroxidation, affects the integrity of the sperm plasma membrane which has high levels of polyunsaturated fatty acids, and decreases sperm quality, affecting their motility and morphology. L-Carnitine exerts its effects by scavenging ROS and reducing oxidative stress. It achieves this by enhancing the expression and activity of enzymes with antioxidant properties, including catalase, glutathione peroxidase, and dismutase. These mechanisms decrease lipid peroxidation, leading to improvements in sperm parameters such as motility, morphology, and concentration. Additionally, L-Carnitine helps to mitigate DNA damage, resulting in an improved fertility rate.

L-Carnitine is a popular supplement often used by athletes for its antioxidant properties, which can enhance performance and aid in post-exercise recovery. Additionally, it is often recognized as a “fat burner” due to its involvement in aerobic metabolism within the mitochondria, promoting fat oxidation and supporting muscle mass [23,24]. Furthermore, L-Carnitine has garnered attention in various therapeutic applications, including its potential role in the treatment of male infertility. Given its involvement in energy metabolism, researchers have investigated L-Carnitine as a potential strategy to improve fertility in men [28,33]. Mitochondria are both the major source of ROS production and a target for OS. By enhancing mitochondrial function in fatty acid transport and metabolism, L-Carnitine scavenges free radicals, minimizing ROS production. Consequently, it may have a positive impact on sperm quality, improving male reproductive outcomes [34]. The beneficial effects of L-Carnitine can be achieved through various pathways. ROS can lead to lipid peroxidation, a process where free radicals attack and damage lipids in cell membranes. In vivo studies have demonstrated that L-Carnitine can reduce the concentration of MDA, a marker of lipid peroxidation, ensuring efficient fatty acid oxidation and preventing the accumulation of damaged lipids [35,36]. Additionally, L-Carnitine is also involved in maintaining the balance of the cellular redox state. It has been shown to enhance the activity of other antioxidants, such as glutathione, which is important in the cellular defense against OS, and promote the expression of enzymes with antioxidant properties when the testis is exposed to toxicants that induce oxidative stress [33]. Enzymes that play a crucial role in protecting cells against ROS include glutathione peroxidase, which facilitates the reduction of hydrogen peroxide and organic peroxides, superoxide dismutase (SOD), which helps in the conversion of superoxide radicals into hydrogen peroxide, and catalase (CAT), which helps in maintaining optimal levels of hydrogen peroxide within cells by neutralizing it. Notably, L-Carnitine has been found to positively influence the expression and activity of these enzymes, thereby supporting, and stimulating the antioxidant defense system [33,37]. Furthermore, L-Carnitine has been associated with an increase in glial cell-derived neurotrophic factor (GDNF), a protein that plays a relevant role in spermatogonial self-renewal [38]. This suggests that L-Carnitine may have a supportive effect on the proliferation and maintenance of spermatogonia, which are vital for continuous sperm production. Considering the multitude of effects that L-Carnitine has in protecting against OS, it may be associated with potential benefits for male fertility. Specifically, L-Carnitine has been shown to have positive effects on improving sperm quality, motility, count, and morphology [39]. Additionally, it plays a role in protecting spermatozoa from DNA damage and preserving acrosome integrity, which is essential for successful fertilization [35]. These combined effects contribute to an overall increase in fertility rates [40,41] (Figure 1). By enhancing the antioxidant defense system, L-Carnitine improves the combat against OS-induced damage, which is known to impair sperm function and overall reproductive success. Its protective actions on spermatozoa can lead to improvements in key parameters related to male fertility, ultimately increasing the chances of successful conception. However, it is important to note that individual responses to L-Carnitine supplementation may vary, and further research is needed to fully understand the optimal dose, duration, and specific populations that may benefit most from its sparse or continuous use [42]. 

This comprehensive review delves into the multifaceted roles of L-Carnitine, a popular supplement among athletes, and its potential to improve sperm quality through its ability to scavenge ROS and mitigate their detrimental effects. The review starts by examining the various functions and signaling pathways associated with L-Carnitine. It then explores findings from in vitro studies conducted on Sertoli cells, in vivo studies involving animal models, and clinical research conducted on infertile men to assess the effects of L-Carnitine on male reproductive health. By providing a thorough review analysis of existing publications, this review aims to shed light on the mechanisms by which L-Carnitine may (positively) influence sperm quality. By elucidating the current knowledge and research gaps, this review contributes to the existing body of literature and may pave the way for further investigation and the development of targeted interventions to optimize male reproductive health. 

## 2. L-Carnitine Is Commonly Used as a Supplement for Professional Athletes: Is It Safe?

In the early 1980s, research began to be performed to study the effects of L-Carnitine on metabolism during exercise, primarily due to its involvement in the β-oxidation of fatty acids that contributes to energy production. L-Carnitine plays a crucial role in facilitating the transport of fatty acids into the mitochondria, where they are oxidized to generate ATP, the body’s main energy currency. Furthermore, L-Carnitine also regulates the pool of acetyl-CoA, a key intermediary in energy metabolism processes [43]. By enhancing fatty acid oxidation and modulating acetyl-CoA levels, L-Carnitine has been hypothesized to have potential benefits in supporting exercise performance and optimizing energy utilization. Research in this field has sought to elucidate the mechanisms underlying the effects of L-Carnitine on metabolism and its potential applications in athletes and individuals engaged in physical activities. To date, numerous studies conducted with athletes have provided valuable insights into the effects of L-Carnitine supplementation. These studies reported a range of benefits through increasing muscle levels of L-Carnitine and preventing its depletion during exercise [44]. Additionally, L-Carnitine supplementation has been associated with improved plasma glucose and ammonia levels, as well as reduced heart rate during exercise [45]. Moreover, L-Carnitine has been shown to enhance performance by improving stress-induced efforts and exerting a positive influence on overall athletic performance [46]. Furthermore, L-Carnitine supplementation has demonstrated a protective effect against exercise-induced muscle damage, leading to enhanced recovery [45,46,47]. In a study by Dragan et al. (1989) [48], the effects of L-Carnitine supplementation were examined in a single-dose group and a 3-week treatment group. The single-dose group experience beneficial effects regarding physical performance, lipid metabolism, muscular function, and lactate accumulation after exercise. On the other hand, the 3-week treatment group experienced positive effects regarding lipid metabolism, evoked muscle potential, and maximal oxygen uptake, leading to better athletic performance. These findings suggest that L-Carnitine may serve as an effective ergogenic aid for professional athletes, with potentially greater benefits in endurance and strength sports. Interestingly, some studies have also investigated the use of L-Carnitine in non-professional athletes across a wide age range, including individuals between the ages of 18 and 50, as well as elderly individuals. These studies have shown that L-Carnitine supplementation can increase serum carnitine concentrations [49] and improve the ability to combat mental and physical fatigue [50]. Taken together, these findings suggest that L-Carnitine supplementation has the potential to enhance performance and combat fatigue in professional and non-professional athletes engaged in various sports and age groups, providing ergogenic benefits, and promoting overall well-being. Furthermore, it raises intriguing possibilities regarding the potential influence of L-Carnitine on male fertility and infertility concerns. It would be interesting to study the relationship between athletes who incorporate L-Carnitine supplementation and their semen quality and fertility outcomes compared to non-athlete men without any supplementation. 

## 3. Molecular Mechanisms of L-Carnitine Function

**Figure 2:**
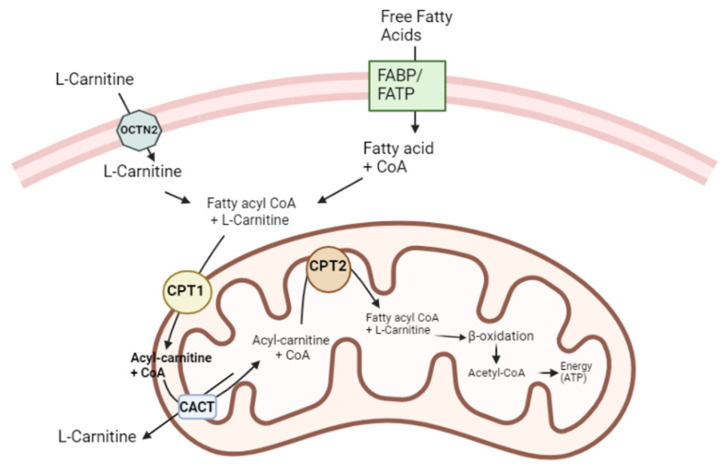
L-Carnitine functions as a cofactor for long-chain fatty acids across the inner mitochondrial membrane. Fatty acids rely on a set of proteins, including transport proteins, to enter the cell. Once inside, long-chain fatty acids bind to CoA to form acyl-CoA. However, the impermeability of the mitochondrial membrane to acyl-CoA requires the involvement of L-Carnitine as a shuttle for the transport of fatty acids into the mitochondria. OCTN2 facilitates the transport of L-Carnitine into the cell. Subsequently, CPT I enables the transfer of the acyl group from acyl-CoA to carnitine, forming acyl-carnitine. CACT then translocates acyl-carnitine across the inner mitochondrial membrane. Once inside the mitochondria, CPT II cleaves acyl-carnitine, releasing the acyl group, which subsequently binds to CoA, initiating β-oxidation. Upon completion of this process, carnitine is transported back to the cytoplasm through the CACT, allowing for its reuse in subsequent cycles. This intricate transport mechanism involving L-Carnitine is essential for the efficient utilization of fatty acids as an energy source within the mitochondria. It ensures the proper delivery of fatty acids to the site of β-oxidation, facilitating their effective metabolism and energy production.

L-Carnitine plays a crucial role in the transport of long-chain fatty acids across the inner mitochondrial membrane [51] (Figure 2). Fatty acids can be produced endogenously or obtained from the blood, and before entering cells, they bind to albumin [52,53]. For fatty acids to enter a cell, a series of proteins are involved in the transport of fatty acids, including fatty acid-binding proteins (FABPpm), fatty acid transport proteins (FATPs), fatty acid translocase (FAT/CD36), and caveolin-1 [54]. To enter mitochondria, long-chain fatty acids must be activated by binding to CoA, which originates acyl-CoA. Notwithstanding, since the mitochondrial membrane is impermeable to acyl-CoA, to enter the mitochondria, fatty acids must resort to a specific enzymatic mechanism that uses L-Carnitine as a carrier molecule [28]. Two important enzymes, carnitine palmitoyl transferase I (CPT I) and carnitine palmitoyl transferase II (CPT II), facilitate the transfer of acyl groups between acyl-CoA and carnitine. CPT I is located on the outer mitochondrial membrane, while CPT II is found on the inner mitochondrial membrane. The organic cation/carnitine transporter 2 (OCTN2) regulates the uptake and distribution of L-Carnitine, and the carnitine acylcarnitine translocase (CACT) assists in the translocation of acyl-carnitine across the inner mitochondrial membrane [24]. In detail, L-Carnitine is transported into a cell by OCTN2. CPT I transfers the acyl group from acyl-CoA to carnitine, forming acyl-carnitine. CACT then transports acyl-carnitine into the mitochondria. Within mitochondria, CPT II cleaves acyl-carnitine, releasing the acyl group, which binds to CoA to initiate β-oxidation. This process generates acetyl-CoA for oxidative phosphorylation or ketone body production in the liver. L-Carnitine returns to the cytoplasm as L-acetyl-carnitine through the CACT for reuse in another cycle [28,53]. The malfunction of this mechanism, especially during fasting, can lead to the release of fat from adipose tissue and its accumulation in the liver, skeletal muscle, and heart [24]. It has been reported that L-Carnitine increases CPT I and CPT II activity [55,56]. However, the exact mechanisms of L-Carnitine’s interaction with these enzymes are not fully understood and need further research. A detailed understanding of this process sheds light on the importance of L-Carnitine in fatty acid metabolism and energy production. 

L-Carnitine also plays a role in maintaining membrane integrity by regulating the acetyl-CoA/CoA ratio within mitochondria [57]. During physical exercise, there is an increase in the acetyl-carnitine/carnitine ratio in muscles, blood, and urine. This elevation helps to maintain higher levels of free coenzyme A and lower levels of acetyl-CoA in tissues [58]. Siliprandi et al. [59] reported that the administration of carnitine alongside physical exercise stimulates the activity of pyruvate dehydrogenase, limited by the availability of NAD+. This stimulation leads to a decrease in lactate and pyruvate levels in plasma and to an increase in acetyl-carnitine levels. Consequently, exercise performance improves, and pyruvate is used more efficiently. This buffering mechanism is particularly important for the oxidation of pyruvate and α-ketoglutarate [60]. During β-oxidation, the breakdown of fatty acids can produce potentially toxic metabolites. L-Carnitine is important in the elimination of these metabolites by binding to acyl residues derived from the intermediary metabolism of amino acids, thereby helping in their removal [61]. Furthermore, L-Carnitine is known for its ability to maintain membrane stability. Its tri-methylamino group and carboxylic group enable interactions with the corresponding poles in membrane phospholipids, glycolipids, and proteins. These interactions help to stabilize the membrane structure and ensure its proper functioning. These functions of L-Carnitine highlight its importance in maintaining cellular homeostasis and preventing the accumulation of toxic metabolites. Additionally, its role in membrane stability underscores its significance in supporting the overall integrity and functionality of cell membranes. This influence is potentially mediated through its impact on the acetylation of membrane phospholipids. Additionally, its amphiphilic properties enable interactions with surface charges present on the cell membrane, potentially contributing to the stabilization of membranes [62,63]. 

The literature also highlights the importance of L-Carnitine in apoptosis. Interestingly, several researchers have questioned whether L-Carnitine exhibits anti-apoptotic properties in various tissues, including skeletal muscle [64], the brain [65], and potentially in male fertility [66,67]. Apoptosis is a natural biological process that occurs during normal cell development to maintain cell homeostasis but it can also be triggered by toxic agents such as ROS. The regulation of apoptosis involves various factors, including BCl-2, which acts as an anti-apoptotic protein, helping to maintain the integrity of the mitochondrial membrane by binding to receptors on the outer mitochondrial membrane; and Bax, which promotes apoptosis by altering the permeability of the outer mitochondrial membrane when a cell is exposed to apoptosis-inducing agents. These alterations release other apoptosis-inducing factors from mitochondria and lead to DNA fragmentation. There have been reports that the function of BCL-2 and Bax genes leads to defects in spermatogenesis and infertility in mice [68,69]. In vivo studies reported that treatment with L-Carnitine increases the expression of BCl-2 and reduces Bax expression in situations where spermatogenesis is compromised, leading to apoptosis in the testes and decreased testosterone secretion [69]. A study also explored the potential anti-apoptotic impact of L-carnitine on the testicular tissue of mice exposed to gamma radiation. The findings of their research revealed that L-Carnitine effectively suppressed the apoptosis of germ cells in the context of radiation therapy [70]. In skeletal muscle, L-Carnitine supplementation has also been shown to inhibit caspases, involved in the apoptotic process, and decrease levels of Tumor Necrosis Factor α (TNF-α) [64]. Caspases, a group of proteases, are fundamental in the initiation and execution of apoptosis. When cells receive death signals, caspases are activated, resulting in morphological changes, including DNA fragmentation [71]. Studies have reported that L-Carnitine protects cells against apoptosis and inhibits caspase activity. While the precise mechanisms underlying the anti-apoptotic effects of L-Carnitine require further investigation, existing findings suggest its potential as an effective anti-apoptotic agent. More research is needed to unravel the intricacies of its mechanisms and explore its therapeutic potential in the prevention of apoptosis-related conditions, especially in the male reproductive system.

## 4. Signaling Pathways Affected by L-Carnitine

Extensive research has focused on elucidating the signaling pathways associated with L-Carnitine, highlighting the involvement of Sirtuin 3 (SIRT3) [72] and nuclear factor erythroid 2-related factor 2 (Nrf2) [73]. SIRT3 is a member of the highly conserved Sirtuin family, having NAD+-dependent protein deacetylase activity in mammals [74]. It predominantly resides in mitochondria, where it exerts control over mitochondrial acetylation and plays a critical role in regulation of metabolic and antioxidant functions [62]. The inhibition of SIRT3 leads to the downregulation of superoxide dismutase 2 (SOD2) expression, resulting in increased levels of ROS and subsequent mitochondrial dysfunction. Reduced levels of SIRT3 have been associated with OS and mitochondrial damage [75]. However, one study demonstrated that treatment with acetyl-L-Carnitine restored the expression and activity of SIRT3, consequently improving mitochondrial function [74]. The precise mechanism underlying the relationship between L-Carnitine and SIRT3 remains unclear, although one hypothesis suggests that the increase in NAD+ induced by L-Carnitine could enhance the activity of NAD+-dependent SIRT3 [76]. NAD+ serves as a substrate for the deacetylation reactions catalyzed by SIRT3. When a cell’s energy status is high, NAD+ levels tend to be elevated, promoting the activation of SIRT3. In summary, by modulating the activity of enzymes such as SOD and CAT, SIRT3 helps to maintain the balance between production and elimination of ROS [77], thus preserving the integrity and functionality of sperm cells. L-Carnitine is also linked to the NRF2 transcription factor, important in the elimination of ROS and the reduction of OS [78]. Under normal conditions, NRF2 is bound to Kelch-like ECH-associated protein 1 (Keap1) in the cytosol. Keap1 functions as a sensor for OS; when cells are exposed to this stimulus, the NRF2–Keap1 complex is disrupted, allowing NRF2 to escape and resulting in Keap1-mediated degradation. As a result, NRF2 accumulates in the cytoplasm. NRF2 then translocates to the nucleus, where it binds to the antioxidant response element (ARE) and acts as a co-transcription factor for various proteins with antioxidant properties. Among the proteins activated by NRF2 are SOD, haem oxygenase-1 (HO-1), involved in the defense system against OS, and γ-glutamyl cysteine synthetase (γ-GCS), a rate-limiting enzyme in glutathione biosynthesis. The products of these NRF2-regulated genes contribute to cellular defense mechanisms against OS and toxins [79]. In a study by Cao et al. [73], L-Carnitine treatment led to increased levels of NRF2, HO-1, and γ-GCS, while Keap1 levels decreased. These findings suggest that the antioxidant effect of L-Carnitine treatment may be mediated through the NRF2–Keap1 pathway. This L-Carnitine–NRF2 interaction highlights the potential of L-Carnitine in modulating cellular antioxidant defense mechanisms and reducing OS. By enhancing the NRF2 activity and the expression of antioxidant proteins, L-Carnitine contributes to the protection of cells against OS and promotes overall cellular health. Additionally, Nrf2 activation may enhance the expression of genes involved in spermatogenesis, sperm motility, and DNA repair, further contributing to male reproductive health [80,81].

## 5. L-Carnitine in Male Fertility 

**Figure 3:**
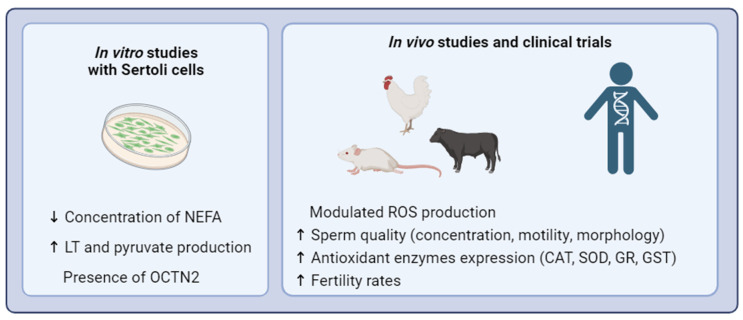
Potential benefits of L-Carnitine supplementation for male fertility In vitro studies with Sertoli cells suggest that L-Carnitine has a role in the metabolism—regulating other functions in these cells related to germ cell nutrition, which leads to an improvement in semen quality. Consistently, clinical trials with infertile men and in vivo studies report that when given L-Carnitine supplementation, there is an increase in sperm parameters, such as concentration, motility, and morphology, a modulation of ROS production, and an increase in antioxidant enzyme expression, such as SOD and CAT. Collectively, these findings suggest that L-Carnitine possesses the potential to wield favorable effects on male infertility. Further exploration is necessary to unravel the intricate mechanisms that underlie these transformative effects. NEFA—Non-esterified fatty acid; LT—Lactate; CAT—Catalase; SOD—Superoxide dismutase; GR—Glutathione Reductase; GST—Glutathione S-Transferase.

The antioxidant properties of L-Carnitine and its role in energy metabolism have become a subject of extensive research for potential therapeutic applications. Various studies have explored its benefits in cardiovascular diseases [82], neuroprotection [83], immune system function [84], and male infertility (Figure 3) [85]. When treated with L-Carnitine, improvements in sperm motility and viability were observed [86]. Male reproductive tissues, such as the epididymis and testis [31,87], exhibit higher concentrations of L-Carnitine compared to plasma, highlighting its relevance to sperm maturation [88] and motility [89]. The transport of free carnitine occurs via passive diffusion across the sperm plasma membrane, with subsequent concentration in the epididymis, particularly in the caudal zone [87]. Eventually, L-Carnitine enters spermatozoa, where it undergoes acetylation upon maturation [75]. Vicardi et al. (2001) [86] reported that L-Carnitine, acting as a cofactor in fatty acid transport, may have a reparative effect by eliminating elevated intracellular levels of toxic acetyl-coenzyme A or by replacing fatty acids in the phospholipid membrane. These findings emphasize the crucial role of L-Carnitine in male reproductive health, suggesting its potential as a therapeutic intervention to enhance fertility. By regulating fatty acid transport, L-Carnitine contributes to the proper functioning and integrity of sperm, providing a favorable environment for sperm maturation, motility, and overall reproductive function.

### 5.1. In Vitro Studies

The effect of L-Carnitine on Sertoli cells, which play a crucial role in spermatogenesis and create a suitable environment within the seminiferous tubules, has been investigated by some researchers, demonstrating a direct action of carnitine on the intermediary metabolism in Sertoli Cells, including Palmero et al. (2000) [90], Caviglia et al. (2004) [91], and Kobayashi et al. (2005) [92]. Sertoli cells need to be very active metabolically, because of their putative metabolic and energetic support to differentiating germ cells. Oxidation of fatty acids is a major energy source for cultured Sertoli cells [6]. Palmero et al. (2000) [90] aimed to explore the metabolic effects of L-Carnitine on Sertoli cells. Their findings demonstrated that exposure to 100 mM of L-Carnitine resulted in a decrease in the concentration of non-esterified fatty acids in Sertoli cells after 24 h, which resulted in an improvement in lipid oxidation. Additionally, L-Carnitine supplementation led to increased production of lactate and pyruvate, enhanced activity of lactate dehydrogenase (LDH), and improved hexose transport, concluding that carnitine may play a crucial role in the intermediary metabolism of Sertoli cells by stimulating lipolytic and glycolytic processes. Consequently, Sertoli cells emerge as potential recipients of carnitine’s metabolic effects within the testicular context. Caviglia et al. (2004) [91] studied the role of carnitines at the testicular level by examining their effects on protein metabolism in rat Sertoli cells. These cells were exposed for 24 h to L-Carnitine, acetyl-L-Carnitine, or both at concentrations ranging from 50–200 mM. They reported that the administration of L-Carnitine and acetyl-L-Carnitine affects the expression of proteins involved in the regulation of glucose uptake, increasing the expression of the membrane glucose transporter-1 and insulin-like growth factor biological activity; the results also showed decreased expression of the negative modulator of the insulin-like growth factor-binding protein. In addition, acetyl-L-Carnitine administration affects protein synthesis, increasing the intracellular amino acid pool useful in speeding up the energy metabolism of Sertoli cells. Taken together, these results suggest a strong probability that carnitine directly influences the somatic cells of the seminiferous epithelium, affecting the development of germ cells. These potential actions of L-Carnitine on Sertoli cells highlight its potential beneficial impact on these cells. Furthermore, Kobayashi et al. (2005) [92] revealed the presence of the carnitine transporter OCTN2 in Sertoli cells and whole-testis tissue samples evaluated by RT-PCR and Western blotting analysis. They observed that carnitine uptake across the basolateral membrane of Sertoli cells is mediated by OCTN2, facilitating the transport of carnitine through the blood–brain barrier from the blood to these cells. The study also investigated the ability of carnitine absorbed by Sertoli cells to cross over to the apical side of these cells. The results suggested that the carnitine taken up by Sertoli cells is transferred to the seminiferous tubular fluid across the blood–testis barrier (BTB). Additionally, the researchers studied M-CPT I and L-CPT I, which are pivotal enzymes in fatty acid oxidation, present in rat and mouse testes. Intriguingly, both Sertoli cells and germ cells express L-CPT I and M-CPT I, implying that carnitine might travel from the bloodstream to Sertoli cells and germ cells. While these studies provide valuable insights, many questions regarding the effects of L-Carnitine on testis function remain unanswered. Future research may explore whether the beneficial effects of L-Carnitine extend to protection against germ cell apoptosis and investigate the capacity of epididymal tissue for carnitine biosynthesis. Addressing these knowledge gaps will further enhance our understanding of the impact of L-Carnitine on Sertoli cells and its potential implications for testicular function and male reproductive health.

### 5.2. In Vivo Studies 

Numerous in vivo studies have investigated the impact of L-Carnitine in animal models. These studies collectively highlight L-Carnitine as a promising therapeutic agent in the treatment of male infertility. A summary of the findings of these studies can be found in Table 1. In a study conducted by M. Khushboo et al. (2018) [93], researchers aimed to determine whether L-Carnitine therapy could prevent the detrimental effects of long-term copper consumption on sperm quality and testis function in Wistar albino rats. The experimental group was administered copper (II) sulfate (CuSO_4_) at a dose of 200 mg/kg along with L-Carnitine at doses of 50 and 100 mg/kg orally for 30 days. The results demonstrated that supplementation with L-Carnitine reduced OS and mitigated the adverse effects of copper. It effectively revitalized sperm quality, restored histological alterations, and rejuvenated spermatogenesis, making it a potential therapeutic agent for copper-induced toxicity. In another study, the protective effect of L-Carnitine on Sertoli cells in the testes against chemotherapy-induced damage was investigated in healthy male adult Kunming mice. Mice were injected with Cyclophosphamide (CTX), a chemotherapeutic alkylating agent known to cause testicular weight loss, transient oligospermia, decreased DNA synthesis in spermatogonia, and protein synthesis in spermatids. Mice also received 100 mg/kg of L-Carnitine once a day for 5 days. The expression of occludin, a protein involved in maintaining tight junction barrier properties, glial cell-derived neurotrophic factor (GDNF), associated with spermatogonial self-renewal, and transforming growth factor-beta (TGF-β), implicated in injury repair processes, were analyzed in Sertoli cells. These proteins play important roles in spermiogenesis, and their secretion by Sertoli cells is crucial for proper testicular function. Results demonstrated higher expression of occludin and GDNF, along with lower expression of TGF-β3, in the group treated with L-Carnitine and CTX compared to the group treated with CTX alone. These findings suggest that L-Carnitine supplementation may enhance antioxidant properties, reverse damage caused by chemotherapy in the testes, and contribute to the recovery of sperm count and motility. These studies provide valuable insights into the protective effects of L-Carnitine in mitigating the adverse effects of copper toxicity and chemotherapy-induced damage on testicular function and sperm quality. These findings highlight the potential therapeutic role of L-Carnitine in preserving male reproductive health under challenging conditions [38]. To investigate the potential role of L-Carnitine against cadmium (Cd), a type I carcinogen known to impair male fertility, a group of mature adult male albino mice were divided into seven groups. All groups were treated intraperitoneally for 30 successive days and were divided as follows: (1) control-received vehicle; (2) treated with cadmium chloride (CdCl_2_) (0.35 mg/kg); (3) treated with Selenium (0.87 mg(kg); (4) treated with L-Carnitine (10 mg/kg); (5) treated with CdCl_2_ + Selenium; (6) treated with CdCl_2_ + L-Carnitine. The study focused on evaluating the activities of CAT, Glutathione Reductase (GR), Superoxide Dismutase (SOD), and Glutathione-S-Transferase (GST)—enzymes relevant for maintaining a well-developed male reproductive system and involved in counteracting the effects of ROS. The findings validated the protective effect of L-Carnitine, suggesting that it may modulate DNA damage, cell proliferation, and chromosomal damage induced by OS [33]. Karam et al. (2022) [94] investigated the effects of L-Carnitine on spermatogenesis using a rat model of hyperlipidemia-induced partial infertility, where testicular tissue damage, and irregular steroidogenesis, occur, among other phenomena. For this, rats were randomly divided into the following three groups: (1) control group; (2) cholesterol feeding group 1, fed 1.5% cholesterol with diet for one month; and (3) cholesterol feeding group 2, fed 1.5% cholesterol with diet + 150 mg/kg body weight L-Carnitine given by water for one month. The results were consistent with previous findings, demonstrating histological protection of the spermatogenic layers in the L-Carnitine-treated group. Moreover, the L-Carnitine treatment group exhibited improved sperm concentration and reduced sperm abnormalities, indicating the potential of L-Carnitine to ameliorate the negative effects of hyperlipidemia on male reproductive health. These studies provide further evidence of the protective role of L-Carnitine against the harmful effects of carcinogens, such as cadmium, and hyperlipidemia-induced infertility. L-Carnitine shows promise in preserving testicular function, spermatogenesis, and overall sperm quality under challenging conditions, suggesting its potential as a therapeutic intervention for male fertility disorders caused by these factors. Heavy metal poisoning is another major concern. Lead, one of the metals that cause death and disease, can originate from a contaminated environment. This can be one of the causes that leads to male infertility. To investigate the effects of L-Carnitine on the mechanisms of reproductive toxicity by induced chronic lead acetate, adult male Wistar rats were treated by oral gavage for 40 days and divided into the following three groups: (1) negative control group; (2) positive control group with 50 mg/kg lead acetate; and (3) group treated with 50 mg/kg lead acetate + 100 mg/kg L-Carnitine. Results reported that chronic lead acetate exposure negatively affected sperm parameters. Additionally, 100 mg/kg of L-Carnitine supplementation improved sperm parameters, testicular OS, hormone, and enzyme levels when compared to the positive control group, indicating that this antioxidant is expected to improve lead acetate-induced male reproductive toxicity [95]. A study was conducted to investigate the effects of dietary supplementation of 50 and 100 mg/kg of L-Carnitine on semen quality, seminal antioxidant activity, and fertility in aged roosters over a 12-week period. The results demonstrated that both dietary concentrations of L-Carnitine significantly improved sperm quality and, subsequently, male fertility. In addition, more studies have found a reduction in MDA, a marker of cellular peroxidation of polyunsaturated fatty acids [37], indicating reduced oxidative damage. Moreover, the activity of seminal CAT and Glutathione Peroxidase (GSH-PX), both crucial indicators of seminal antioxidant efficiency, increased in the treated groups. These findings highlight the role of L-Carnitine in enhancing seminal antioxidant activity, preventing MDA production, and protecting spermatozoa during storage in the epididymis and oviduct in aged roosters. 

Furthermore, studies conducted with buffalo semen samples cryopreserved with 2.5 and 7.5 mM carnitine [96] and human semen samples cryopreserved with L-Carnitine at 5 mmol/L (established optimal concentration) [97] also demonstrated L-Carnitine’s beneficial effects in preserving sperm quality and enhancing motility during cryopreservation. L-Carnitine has been found to increase ATP generation and modulate ROS production, contributing to improved sperm parameters [96,97]. Overall, the data suggest that 50 or 100 mg/kg of L-Carnitine in male Wistar rats has a potential therapeutic effect in improving male fertility, reducing damage caused by ROS, and enhancing various sperm parameters, including motility, viability, concentration, and morphology. Additionally, L-Carnitine exhibits protective properties, such as histological protection for spermatogenic layers, and enhances the functions of Sertoli and Leydig cells. These findings highlight the potential of L-Carnitine as a valuable supplement for enhancing male reproductive health.

**Table 1:** Effects of L-Carnitine in vivo on male reproductive function.

Aims of the Study	Period, Dosing, Route of Administration	Model	Main Results	References
Examine whether the detrimental effects of long-term copper consumption on sperm quality and testis function of Wistar albino rats could be prevented by LC therapy	30 days; 200 mg/kg CuSO_4_ 50 and 100 mg/kg LC; oral administration	3-month-old Wistar rats	Revitalization of sperm quality (motility, viability, number); Restoration of histological alterations (germ cell depletion, sloughing of germ cells, vacuolization, and degeneration); Rejuvenation of spermatogenesis.	M. Khushboo et al., 2017 [93]
Study the protective effect of LC on Sertoli testis cells from the damage of chemotherapy	5 days; 100 mg/kg LC intraperitoneally administration	Testis Sertoli cells from adult male mice	Recovery of the sperm count and sperm motility; Higher expressions of occluding and GDNF; Lower expression of TGF-β3.	Y. Cao et al., 2017 [38]
Evaluate the effect of carnitine supplementation of semen extender on fertility parameters of frozen-thawed buffalo sperm	2.5 and 7.5 mM carnitine	Cryopreserved Buffalo semen	Increased ATP generation; Modulated ROS production.	V. Longobardi et al., 2017 [96]
Evaluated whether some spermatic qualitative parameters could be ameliorated by carnitine treatment in adult rats exposed to doxorubicin	A single dose of LC (250 mg/kg body weight)	Pre-pubertal male Wistar rats	Increase in intact acrosome integrity; Decreased MDA and nitrite concentration; Increased fertility and implantation rate; Decreased spermatozoa with damaged DNA.	R. Cabral et al., 2017 [35]
Investigate the protective effect of LC and L-arginine on semen quality, OS parameters, and testis cell energy after busulfan treatment	single I.P. injection of busulfan; 1 mL of L-arginine daily by oral gavage; 1 mL of LC by oral gavages	Adult male Wistar rats	Improved sperm morphology, motility, velocity, and count; Increased MDA and ATP.	Abd-Elrazek et al., 2017 [98]
Investigate the effects of dietary LC on semen quality, seminal antioxidant activity, and their implications for fertility in aged roosters	12 weeks; 50 and 150 mg/kg body weight/day of LC	Aged roosters	Increased sperm quality; Increased plasma concentration of testosterone; Increased seminal MDA concentration, CAT, and GSH-PX activity.	Elokil et al., 2019 [37]
Investigate the possible protective role of Se and LC against the adverse effects induced by cadmium	30 days; LC at a dose of 10 mg/kg	Mature adult male albino mice	Increased CAT, GR, SOD, and GST activities; Less histopathological abnormalities; Less DNA damage.	Alharthi et al., 2020 [33]
Investigate the possible effect of LC on the mechanisms of reproductive toxicity induced by chronic lead acetate treatment	40 days by oral gavage; 50 mg/kg of lead acetate; 100 mg/kg of LC	Male Wistar rats	Reduced testicular OS; Improved sperm parameters; Elevated serum FSH, LH, and testosterone.	Abdel-Emam et al., 2021 [95]
Evaluate the underlying mechanism of the ameliorative effects of LC	30 days; diet supplemented with 1.5% cholesterol + LC 150 mg/kg given orally	Premature albino male rats	Histological protection for spermatogenic layers; Better concentration of sperm; Reduced sperm abnormalities.	Karam et al., 2022 [94]

Abbreviations: LC—L-Carnitine; CuSO_4_—Copper(II) sulfate; GDNF—Glial-derived neurotrophic factor; TGF-β3—Transforming growth factor-β3; ROS—Reactive Oxygen Species; MDA—malondialdehyde; CAT—catalase; GSH-PX—Glutathione peroxidase; GR—Glutathione reductase; SOD—superoxide dismutase; GST—Glutathione S-Transferase; OS—oxidative stress; FSH—Follicle-Stimulating Hormone; LH—Luteinizing Hormone.

### 5.3. Clinical Studies 

Studies investigating the effects of L-Carnitine on infertile men demonstrate that L-Carnitine supplementation can improve sperm parameters. Moreover, L-Carnitine has shown superiority over other antioxidants such as vitamin E and CoQ10 in terms of improving sperm quality and increasing pregnancy rates [39]. A summary of the findings from these studies can be found in Table 2, providing valuable insights on the effectiveness of L-Carnitine as a therapeutic intervention for male infertility. Khademi et al. (2005) [99] conducted a clinical study to determine the effects of L-Carnitine on sperm parameters in patients with idiopathic sperm abnormalities. An amount of 1 g of L-Carnitine was orally given to patients three times daily for 3 months. A sperm assessment was performed before and after the treatment. The results showed that L-Carnitine improved the percentile of motile spermatozoa, the percentile of grade A sperm, and the percentile of normal-shaped sperm. However, there were no differences in the sperm density. To study the same effects, L.L. Sun et al. (2018) [100] administered 1 g of L-Carnitine three times a day to infertile male patients with low acrosin activity. They used vitamin E (VE) as a control, and despite the fact that sperm concentration and sperm acrosin activity improved in the VE control group after treatment, there was no statistically significant difference. The L-Carnitine-supplemented group showed consistent results, reporting an increase in sperm motility, sperm concentration, and sperm acrosin activity. In another study, patients received treatment twice a day, 15 g/bag at a time, orally. This research aimed to compare L-Carnitine supplementation with Coq10 and vitamin E (used as control). All sperm parameters) and hormone levels (testosterone and LH) showed significant improvement in the L-Carnitine-supplemented group when compared to the baseline. L-Carnitine emerges as the superior choice for improving sperm parameters compared to the combination of CoQ10 and vitamin E. While CoQ10 and vitamin E show positive effects on sperm motility, morphology, and testosterone levels, L-Carnitine goes a step further by enhancing sperm concentration and LH levels, offering a more comprehensive and effective solution for optimizing male reproductive health [39]. Wang et al. (2010) [40] studied patients with asthenozoospermia and treated them with L-Carnitine (2 g/day) for 3 months. They reported an increase in the percentage of motile sperm after treatment with L-Carnitine, but no statistically significant differences were found in sperm density and in the percentage of sperm with normal morphology. Another important issue is the damage caused by cryopreservation of sperm as part of assisted reproductive technology. Most of this damage is caused by ROS. L-Carnitine, due to its antioxidant properties, can improve this process, maintaining semen with higher quality. Results reported that L-Carnitine improved sperm motility, reduced DNA damage, and decreased ROS levels, before and after freezing [101]. Many researchers used L-Carnitine combined with other micronutrients to determine its effects. Lenzi et al. (2004) [28] combined 2 g/day of L-Carnitine with 1 g/day of acetyl-L-Carnitine as a treatment for patients with oligoasthenoteratozoospermia for 6 months. The results reported an increase in all sperm parameters after combined treatment with carnitine. Interestingly, the most significant improvement in sperm motility was present in patients who had lower initial absolute values of motile sperm. It would be interesting if there was a third group to compare a L-Carnitine-only treatment with the combined treatment. 

Additionally, studies using a combined treatment of L-Carnitine, acetyl-L-Carnitine, and other micronutrients once a day for 6 months also showed positive results regarding sperm quality in male infertility patients [23,41,102]. Sperm concentration was significantly increased in supplemented patients compared to the placebo. Total sperm count also increased significantly in the supplemented group compared to the placebo group and sperm motility was higher in supplemented patients [102]. In addition to increased motility, increased morphology has been reported following the third month of therapy. The level of free oxygen radicals decreased in the supplemented group compared to the placebo group, an indicator of OS, and DNA damage was increased in the placebo group compared to supplemented group [23]. Consequently, the benefits of L-Carnitine can improve fertility rates [41]. Based on the results of these clinical studies, we can conclude that L-Carnitine, taken 2 to 10 g/day for 3 to 6 months, has a beneficial effect in male infertile patients or when combined with other micronutrients. 

Overall, all studies show similar results. The optimal dose remains to be established. However, based on these studies, a dose between 1 to 3 g/day of L-Carnitine for a period of 3 to 6 moths improves sperm quality, hormone levels, and decreases ROS levels in infertile men when compared to fertile men. Despite these positive results, it is necessary to explore the mechanisms underlying this process. 

Regarding adverse events, L-Carnitine does not have an established tolerable upper intake. The studies either do not report negative effects or they are not severe and not enough to stop the therapy nor induce nausea, headache, or vertigo [102]. However, doses of more than 3 g/day of carnitine supplements can cause nausea, vomiting, abdominal cramps, diarrhea, and muscle weakness in people with uremia and seizures in those with seizure disorders [103,104]. It is crucial to approach antioxidant supplementation under the guidance of a healthcare professional, taking into consideration individual patient factors. Allergies, medication interactions, and other personal considerations must be carefully evaluated before incorporating these supplements. In addition to L-Carnitine, the potential benefits of antioxidants such as Quercetin [105] and Resveratrol [106] have also been explored in relation to male infertility. These compounds exhibit the potential to alleviate oxidative stress and enhance sperm quality, providing an alternative option for patients who may not benefit from L-Carnitine.

**Table 2:** Effects of L-Carnitine supplementation in men fertility.

Aims of the Study	Period, Dosing, Route of Administration	Population	Main Results	References
Determine the effects of LC on sperm parameters in patients with idiopathic sperm abnormalities	3 months; 1 g of LC orally; 3 times a day	48 smokers and 122 non-smokers (Aged 20–56 years)	Increased sperm motility; Increased normal-shaped spermatozoa.	Khademi et al., 2005 [99]
Explore the clinical effect of LC on infertile males with asthenozoospermia	3 months; 2 g/day of LC	135 patients with asthenozoospermia	Increased sperm motility; Raised rate of pregnancy.	Wang et al., 2010 [40]
Determine the efficacy of combined LC and ALC therapy in infertile males with oligoasthenoteratozoospermia	2 months’ wash out, 6 months’ therapy, 2 month follow-up; 10 mL vial containing 2 g/d carnitine orally administered; 500 mg twice per day orally every 12 h of ALC;	60 infertile men with oligoasthenoteratozoospermia (Aged 20–40 years)	Increased sperm motility.	Lenzi et al., 2004 [28]
Investigate the effect of supplementation with LC and ALC on sperm quality in 104 subjects with oligo- and/or astheno- and/or teratozoospermia with or without varicocele.	6 months; two sachets daily of a supplement containing 1000 mg LC, 500 mg ALC, and other micronutrients	104 men with oligo and/or astheno and/or teratozoospermia with or without varicocele (Aged 18–48 years)	Increased sperm concentration; Increased total sperm count; Increased total motility.	Busetto et al., 2018 [102]
Evaluate the effect of LC on low sperm acrosin activity in infertile men	3 months; 1 g of LC	240 male infertility patients with low sperm acrosin activity	Increased sperm motility; Increased sperm acrosin activity; Increased sperm concentration.	Sun et al., 2018 [100]
Study the effects of complex ALC, LC, fumarate, and alpha-lipoic acid on oxidative stress, ejaculate quality, and sperm DNA fragmentation in men with infertility.	6 months; 10 g of the supplement once a day	80 infertile men with increased levels of sperm DNA fragmentation and oxidative stress (Aged 25–40 years)	Improved sperm mobility and morphology; Decreased free oxygen radicals: Decrease in DNA fragmentation.	Gamidov et al., 2019 [23]
Evaluate a combined treatment with LC and acetyl-L-Carnitine with micronutrients as a treatment for infertility in men	6 months; 1.0 g LC, 0.5 g ALC and other micronutrients	175 males with idiopathic oligoasthenozoospermia (Aged 19–44 years)	Decreased DNA fragmentation; Increased seminal carnitine concentration; Improved sperm motility.	Micic et al., 2018 [107]
Comparing sperm parameters and hormonal levels with LC vs. CoQ10 and vitamin E therapy for patients with asthenozoospermia and teratozoospermia.	3 months; 15 g/bag LC complex; orally, one bag at a time, twice a day	143 patients with asthenozoospermia and teratozoospermia.	Increased sperm motility, morphology, and concentration; Improved testosterone levels; Superior to the others in improving sperm quality.	Ma et al., 2020 [39]
Study the influence of a multi-component nutrient dietary supplement on sperm parameters and pregnancy rates in idiopathic male infertility	6 months; supplement containing LC/ALC, l-arginine, glutathione, co-enzyme Q10, zinc, vitamin B9, vitamin B12, selenium, once daily	83 males aged 21–50 years with oligo-, astheno-, and teratozoospermia (Aged 21–50 years)	Improved sperm quality (concentration, motility, and morphology); Increased fertility rates.	Kopets et al., 2020 [41]
Assess the effect of LC and CoQ10 during, before, and after freezing in oligospermia men	1 h to 2 weeks; G1—LC (100 µM) for 1 h; G3—CoQ10 (100 µM) for 1 h; G4—LC and CoQ10 (100 µM) for 1 h; G5—freezing control group; G6—frozen with CoQ10 (100 µM); G7—frozen with LC (100 µM); G8—frozen with 100 µM (LC + CoQ10) for 2 weeks.	30 oligospermic men (Aged 25–40 years)	Decreased ROS levels; Improved motility and protamine deficiency.	Nezhad et al., 2021 [101]

Abbreviations: LC—L-Carnitine; ALC—acetyl-L-Carnitine; CoQ10—Coenzyme Q10.

## 6. Materials and Methods 

The scientific literature discussed in this review was collected by searching the database PubMed. Preference was given to more recent articles, even though some had to be from an earlier period due to the lack of new information and to build a historical perspective. To identify relevant papers, the following keywords were combined: “L-Carnitine AND male infertility/fertility”; “L-Carnitine AND Athletes”; “L-Carnitine and Sertoli cells/in vitro”; “L-Carnitine AND Sirt3/Nrf2”; “Antioxidants AND male fertility”; “L-Carnitine AND in vivo studies”; “L-Carnitine and mitochondria”; “L-Carnitine AND sperm”; “L-Carnitine AND Reactive Oxygen Species/Oxidative Stress”. Only papers written in English were considered in this review. Relevant articles, at least to some degree, had to examine the relationship between L-Carnitine, mitochondria, and male fertility and the mechanisms and signal pathways of L-Carnitine and had to give details on the effect of L-Carnitine in male (in)fertility. 

## 7. Conclusions and Future Perspectives

L-Carnitine, a natural antioxidant, has gained attention as a potential therapeutic compound for various pathologies, including male infertility. ROS are known to cause DNA damage and OS, leading to sperm damage when cellular antioxidant defenses are overwhelmed. This review discussed the effects of L-Carnitine on male reproductive health, encompassing studies conducted in vitro and in vivo and in human studies. The results obtained so far have been promising, demonstrating that 1 to 3 g/day of L-Carnitine for 3 to 6 months can improve sperm parameters, such as motility, concentration, morphology, and sperm count. Additionally, some studies have reported improved pregnancy rates after men’s supplementation with L-Carnitine. While the findings suggest the potential benefits of L-Carnitine on male fertility, further research is required to elucidate the underlying mechanisms of its therapeutic actions. It is important to explore its effects on specific cells of the male reproductive system, such as Sertoli and Leydig cells, and to investigate the signaling pathways involved in its action. Understanding these mechanisms will provide a deeper understanding of the role of L-Carnitine in male reproductive health and may have the way for more targeted therapeutic interventions. It would also be interesting to study the relationship between athletes using L-Carnitine supplementation and their fertility when compared to non-athletes without supplementation. Overall, L-Carnitine shows promise as a potential therapeutic agent to improve male fertility and address sperm-related issues. Continued research efforts will help refine our understanding of its therapeutic potential and its specific mechanisms of action, leading to better treatment options for individuals experiencing infertility.

## Data Availability

Not applicable.

## References

[1] World Health Organization Infertility. https://www.who.int/news-room/fact-sheets/detail/infertility.

[2] Babakhanzadeh E., Nazari M., Ghasemifar S., Khodadadian A. (2020). Some of the Factors Involved in Male Infertility: A Prospective Review. Int. J. Gen. Med..

[3] Chua M.E., Escusa K.G., Luna S., Tapia L.C., Dofitas B., Morales M. (2013). Revisiting oestrogen antagonists (clomiphene or tamoxifen) as medical empiric therapy for idiopathic male infertility: A meta-analysis. Andrology.

[4] Sharma A., Minhas S., Dhillo W.S., Jayasena C.N. (2021). Male infertility due to testicular disorders. J. Clin. Endocrinol. Metab..

[5] Gilbert S.F. (2000). Spermatogenesis. Developmental Biology.

[6] Cole L.A. (2018). Human Male Spermatogenesis in Encyclopedia of Reproduction.

[7] Rato L., Alves M.G., Socorro S., Duarte A.I., Cavaco J.E., Oliveira P.F. (2012). Metabolic regulation is important for spermatogenesis. Nat. Rev. Urol..

[8] Abou-Haila A., Tulsiani D.R. (2000). Mammalian Sperm Acrosome: Formation, Contents, and Function. Arch. Biochem. Biophys..

[9] Cheng C.Y., Mruk D.D. (2010). The biology of spermatogenesis: The past, present and future. Philos. Trans. R. Soc. B Biol. Sci..

[10] Tremellen K. (2008). Oxidative stress and male infertility—A clinical perspective. Hum. Reprod. Updat..

[11] Martin-Hidalgo D., Bragado M.J., Batista A.R., Oliveira P.F., Alves M.G. (2019). Antioxidants and Male Fertility: From Molecular Studies to Clinical Evidence. Antioxidants.

[12] Wright C., Milne S., Leeson H. (2014). Sperm DNA damage caused by oxidative stress: Modifiable clinical, lifestyle and nutritional factors in male infertility. Reprod. Biomed. Online.

[13] Smith M.A., Perry G., Pryor W.A. (2002). Causes and consequences of oxidative stress in Alzheimer’s disease. Free Radic. Biol. Med..

[14] Halliwell B. (1996). Antioxidants in human health and disease. Annu. Rev. Nutr..

[15] Forman H.J., Ursini F., Maiorino M. (2014). An overview of mechanisms of redox signaling. J. Mol. Cell. Cardiol..

[16] Sakkas D., Mariethoz E., Manicardi G., Bizzaro D., Bianchi P., Bianchi U. (1999). Origin of DNA damage in ejaculated human spermatozoa. Rev. Reprod..

[17] Dutta S., Sengupta P., Slama P., Roychoudhury S. (2021). Oxidative Stress, Testicular Inflammatory Pathways, and Male Reproduction. Int. J. Mol. Sci..

[18] Hosen M.B., Islam M.R., Begum F., Kabir Y., Howlader M.Z.H. (2015). Oxidative Stress Induced Sperm DNA Damage, a Possible Reason for Male Infertility. Iran. J. Reprod. Med..

[19] Gomez E., Irvine D.S., Aitken R.J. (1998). Evaluation of a spectrophotometric assay for the measurement of malondialdehyde and 4-hydroxyalkenals in human spermatozoa: Relationships with semen quality and sperm function. Int. J. Androl..

[20] Cilio S., Rienzo M., Villano G., Mirto B.F., Giampaglia G., Capone F., Ferretti G., Di Zazzo E., Crocetto F. (2022). Beneficial Effects of Antioxidants in Male Infertility Management: A Narrative Review. Oxygen.

[21] Soumya R., Carl H., Vani R. (2016). l-carnitine as a Potential Additive in Blood Storage Solutions: A Study on Erythrocytes. Indian J. Hematol. Blood Transfus..

[22] Ahmadi S., Bashiri R., Ghadiri-Anari A., Nadjarzadeh A. (2016). Antioxidant supplements and semen parameters: An evidence based review. Int. J. Reprod. Biomed..

[23] Gamidov S.G., Ovchinnikov R.O., Popova A.P. (2019). Double-blind, randomized placebo-controlled study of efficiency and safety of complex acetyl-L-carnitine, L-carnitine fumarate and alpha-lipoic acid (Spermactin^®^ forte) for treatment of male infertility. Urologiia.

[24] Longo N., Frigeni M., Pasquali M. (2016). Carnitine transport and fatty acid oxidation. Biochim. Biophys. Acta.

[25] Pekala J., Patkowska-Sokola B., Bodkowski R., Jamroz D., Nowakowski P., Lochynski S., Librowski T. (2011). L-Carnitine—Metabolic Functions and Meaning in Humans Life. Curr. Drug Metab..

[26] Karlic H., Lohninger A. (2004). Supplementation of L-Carnitine in athletes: Does it make sense?. Nutrition.

[27] Adeva-Andany M.M., Calvo-Castro I., Fernández-Fernández C., Donapetry-García C., Pedre-Piñeiro A.M. (2017). Significance of L-Carnitine for Human Health. IUBMB Life.

[28] Lenzi A., Sgro P., Salacone P., Paoli D., Gilio B., Lombardo F., Santulli M., Agarwal A., Gandini L. (2004). A placebo-controlled double-blind randomized trial of the use of combined L-Carnitine and L-acetyl-carnitine treatment in men with asthenozoospermia. Fertil. Steril..

[29] Jeulin C., Lewin L. (1996). Role of free L-Carnitine and acetyl-L-Carnitine in post-gonadal maturation of mammalian spermatozoa. Hum. Reprod. Update.

[30] Mostafa T., Abougabal K., Mintziori G., Nabil N., Adel M., AboSief A.F. (2022). Seminal L-Carnitine In Infertile Oligoasthenoteratozoospermic Men with Varicocele. J. Reprod. Infertil..

[31] Lewin L.M., Beer R., Lunenfeld B. (1976). Epididymis and seminal vesicle as sources of carnitine in human seminal fluid: The clinical significance of the carnitine concentration in human seminal fluid. Fertil. Steril..

[32] Menchini-Fabris G.F., Canale D., Izzo P.L., Olivieri L., Bartelloni M. (1984). Free L-Carnitine in human semen: Its variability in different andrologic pathologies. Fertil. Steril..

[33] Alharthi W.A., Hamza R.Z., Elmahdi M.M., Abuelzahab H.S.H., Saleh H. (2020). Selenium and L-Carnitine Ameliorate Reproductive Toxicity Induced by Cadmium in Male Mice. Biol. Trace Element Res..

[34] Aliabadi E., Mehranjani M.S., Borzoei Z., Talaei-Khozani T., Mirkhani H., Tabesh H. (2012). Effects of L-Carnitine and L-acetyl-carnitine on testicular sperm motility and chromatin quality. Iran. J. Reprod. Med..

[35] Cabral R.E.L., Mendes T.B., Vendramini V., Miraglia S.M. (2018). Carnitine partially improves oxidative stress, acrosome integrity, and reproductive competence in doxorubicin-treated rats. Andrology.

[36] Thangasamy T., Jeyakumar P., Sittadjody S., Joyee A.G., Chinnakannu P. (2009). L-Carnitine mediates protection against DNA damage in lymphocytes of aged rats. Biogerontology.

[37] Elokil A.A., Bhuiyan A.A., Liu H.-Z., Hussein M.N., Ahmed H.I., Azmal S.A., Yang L., Li S. (2019). The capability of L-Carnitine-mediated antioxidant on cock during aging: Evidence for the improved semen quality and enhanced testicular expressions of GnRH1, GnRHR, and melatonin receptors MT 1/2. Poult. Sci..

[38] Cao Y., Wang X., Li S., Wang H., Yu L., Wang P. (2017). The Effects of L-Carnitine against Cyclophosphamide-Induced Injuries in Mouse Testis. Basic Clin. Pharmacol. Toxicol..

[39] Ma L., Sun Y. (2022). Comparison of L-Carnitine vs. Coq10 and Vitamin E for idiopathic male infertility: A randomized controlled trial. Eur. Rev. Med. Pharmacol. Sci..

[40] Wang Y.-X., Yang S.-W., Qu C.-B., Huo H.-X., Li W., Li J.-D., Chang X.-L., Cai G.-Z. (2010). L-Carnitine: Safe and effective for asthenozoospermia. Zhonghua Nan Ke Xue = Natl. J. Androl..

[41] Kopets R., Kuibida I., Chernyavska I., Cherepanyn V., Mazo R., Fedevych V., Gerasymov S. (2020). Dietary supplementation with a novel L-Carnitine multi-micronutrient in idiopathic male subfertility involving oligo-, astheno-, teratozoospermia: A randomized clinical study. Andrology.

[42] Agarwal A., Virk G., Ong C., Du Plessis S.S. (2014). Effect of Oxidative Stress on Male Reproduction. World J. Men’s Health.

[43] Fielding R., Riede L., Lugo J.P., Bellamine A. (2018). l-Carnitine Supplementation in Recovery after Exercise. Nutrients.

[44] Arenas J., Ricoy J.R., Encinas A.R., Pola P., D’Iddio S., Zeviani M., DiDonato S., Corsi M. (1991). Carnitine in muscle, serum, and urine of nonprofessional athletes: Effects of physical exercise, training, and L-Carnitine administration. Muscle Nerve.

[45] Broad E.M., Maughan R.J., Galloway S.D. (2008). Carbohydrate, protein, and fat metabolism during exercise after oral carnitine supplementation in humans. Int. J. Sport Nutr. Exerc. Metab..

[46] Dragan G.I., Pleosteanu E., Selejan V. (1988). Studies concerning the ergogenic value of Cantamega-2000^®^ supply in top junior cyclists. Physiologie.

[47] Giamberardino M.A., Dragani L., Valente R., Di Lisa F., Saggin R., Vecchiet L. (1996). Effects of prolonged L-Carnitine administration on delayed muscle pain and CK release after eccentric effort. Int. J. Sports Med..

[48] DrĂgan I.G., Vasiliu A., Georgescu E., Eremia N. (1989). Studies concerning chronic and acute effects of L-Carnitina in elite athletes. Rev. Roum. Morphol. Physiol.-Ser. Physiol..

[49] Parandak K., Arazi H., Khoshkhahesh F., Nakhostin-Roohi B. (2014). The effect of two-week L-Carnitine supplementation on exercise—Induced oxidative stress and muscle damage. Asian J. Sports Med..

[50] Pistone G., Marino A.D., Leotta C., Dell’Arte S., Finocchiaro G., Malaguarnera M. (2003). Levocarnitine administration in elderly subjects with rapid muscle fatigue: Effect on body composition, lipid profile and fatigue. Drugs Aging.

[51] McGarry J.D., Brown N.F. (1997). The mitochondrial carnitine palmitoyltransferase system: From concept to molecular analysis. Eur. J. Biochem..

[52] Cooper D.E., Young P.A., Klett E.L., Coleman R.A. (2015). Physiological consequences of compartmentalized acyl-CoA metabolism. J. Biol. Chem..

[53] Longo N., di San Filippo C.A., Pasquali M. (2006). Disorders of carnitine transport and the carnitine cycle. Am. J. Med. Genet. Part C Semin. Med. Genet..

[54] Anderson C.M., Stahl A. (2013). SLC27 fatty acid transport proteins. Mol. Asp. Med..

[55] Jain S., Singh S.N. (2015). Effect of L-Carnitine Supplementation on Nutritional Status and Physical Performance Under Calorie Restriction. Indian J. Clin. Biochem..

[56] Yoon H.-R., Hong Y.M., Boriack R.L., Bennett M.J. (2003). Effect of L-Carnitine supplementation on cardiac carnitine palmitoyltransferase activities and plasma carnitine concentrations in adriamycin-treated rats. Pediatr. Res..

[57] Jeukendrup A.E. (2002). Regulation of fat metabolism in skeletal muscle. Ann. N. Y. Acad. Sci..

[58] Marriott B.M., Institute of Medicine (US) Committee on Military Nutrition Research, The Role of Carnitine in Enhancing Physical Performance (1994). Food Components to Enhance Performance: An Evaluation of Potential Performance-Enhancing Food Components for Operational Rations.

[59] Siliprandi N., Di Lisa F., Pieralisi G., Ripari P., Maccari F., Menabo R., Giamberardino M.A., Vecchiat L. (1990). Metabolic changes induced by maximal exercise in human subjects following L-Carnitine administration. Biochim. Biophys. Acta (BBA)—Gen. Subj..

[60] Wanders R.J., Visser G., Ferdinandusse S., Vaz F.M., Houtkooper R.H. (2020). Mitochondrial Fatty Acid Oxidation Disorders: Laboratory Diagnosis, Pathogenesis, and the Complicated Route to Treatment. J. Lipid Atheroscler..

[61] Virmani M.A., Cirulli M. (2022). The Role of l-Carnitine in Mitochondria, Prevention of Metabolic Inflexibility and Disease Initiation. Int. J. Mol. Sci..

[62] Virmani A., Binienda Z. (2004). Role of carnitine esters in brain neuropathology. Mol. Asp. Med..

[63] Hülsmann W., Dubelaar M.-L., Lamers J., Maccari F. (1985). Protection by acyl-carnitines and phenylmethylsulfonyl fluoride of rat heart subjected to ischemia and reperfusion. Biochim. Biophys. Acta (BBA)—Mol. Cell Res..

[64] Vescovo G., Ravara B., Gobbo V., Sandri M., Angelini A., Della Barbera M., Dona M., Peluso G., Calvani M., Mosconi L. (2002). L-Carnitine: A potential treatment for blocking apoptosis and preventing skeletal muscle myopathy in heart failure. Am. J. Physiol. Cell Physiol..

[65] Afshin-Majd S., Bashiri K., Kiasalari Z., Baluchnejadmojarad T., Sedaghat R., Roghani M. (2017). Acetyl-L-carnitine protects dopaminergic nigrostriatal pathway in 6-hydroxydopamine-induced model of Parkinson’s disease in the rat. Biomed. Pharmacother..

[66] Abdelrazik H., Sharma R., Mahfouz R., Agarwal A. (2009). L-Carnitine decreases DNA damage and improves the in vitro blastocyst development rate in mouse embryos. Fertil. Steril..

[67] Cankorkmaz L., Köylüoğlu G., Özer H., Yıldız E., Sümer Z., Özdemir Ö. (2009). The role of apoptosis and protective effect of carnitine in contralateral testicular injury in experimental unilateral testicular torsion. Turk. J. Trauma Emerg. Surg..

[68] Ranger A.M., Malynn B.A., Korsmeyer S.J. (2001). Mouse models of cell death. Nat. Genet..

[69] Vardiyan R., Ezati D., Anvari M., Ghasemi N., Talebi A. (2020). Effect of L-carnitine on the expression of the apoptotic genes Bcl-2 and Bax. Clin. Exp. Reprod. Med..

[70] Altun Z., Olgun Y., Ercetin P., Aktas S., Kirkim G., Serbetcioglu B., Olgun N., Guneri E.A. (2014). Protective effect of acetyl-l-carnitine against cisplatin ototoxicity: Role of apoptosis-related genes and pro-inflammatory cytokines. Cell Prolif..

[71] Mutomba M.C., Yuan H., Konyavko M., Adachi S., Yokoyama C.B., Esser V., McGarry J., Babior B.M., Gottlieb R.A. (2000). Regulation of the activity of caspases by L-carnitine and palmitoylcarnitine. FEBS Lett..

[72] Hirschey M.D., Shimazu T., Goetzman E., Jing E., Schwer B., Lombard D.B., Grueter C.A., Harris C., Biddinger S., Ilkayeva O.R. (2010). SIRT3 regulates mitochondrial fatty-acid oxidation by reversible enzyme deacetylation. Nature.

[73] Cao Y., Li X., Wang C.-J., Li P., Yang B., Wang C.-B., Wang L.-X. (2015). Role of NF-E2-related factor 2 in neuroprotective effect of L-Carnitine against high glucose-induced oxidative stress in the retinal ganglion cells. Biomed. Pharmacother..

[74] Morigi M., Perico L., Rota C., Longaretti L., Conti S., Rottoli D., Novelli R., Remuzzi G., Benigni A. (2015). Sirtuin 3–dependent mitochondrial dynamic improvements protect against acute kidney injury. J. Clin. Investig..

[75] Dikalova A.E., Pandey A., Xiao L., Arslanbaeva L., Sidorova T., Lopez M.G., Billings F.T., Verdin E., Auwerx J., Harrison D.G. (2020). Mitochondrial Deacetylase Sirt3 Reduces Vascular Dysfunction and Hypertension While Sirt3 Depletion in Essential Hypertension Is Linked to Vascular Inflammation and Oxidative Stress. Circ. Res..

[76] Shen Y., Wu Q., Shi J., Zhou S. (2020). Regulation of SIRT3 on mitochondrial functions and oxidative stress in Parkinson’s disease. Biomed. Pharmacother..

[77] Chen J., Chen S., Zhang B., Liu J. (2021). SIRT3 as a potential therapeutic target for heart failure. Pharmacol. Res..

[78] Zhang D.-M., Guo Z.-X., Zhao Y.-L., Wang Q.-J., Gao Y.-S., Yu T., Chen Y.-K., Chen X.-M., Wang G.-Q. (2019). L-Carnitine regulated Nrf2/Keap1 activation in vitro and in vivo and protected oxidized fish oil-induced inflammation response by inhibiting the NF-κB signaling pathway in *Rhynchocypris lagowski* Dybowski. Fish Shellfish. Immunol..

[79] Nguyen T., Nioi P., Pickett C.B. (2009). The Nrf2-Antioxidant Response Element Signaling Pathway and Its Activation by Oxidative Stress. J. Biol. Chem..

[80] Aydos O.S., Yukselten Y., Aydos D., Sunguroglu A., Aydos K. (2021). Relationship between functional Nrf2 gene promoter polymorphism and sperm DNA damage in male infertility. Syst. Biol. Reprod. Med..

[81] Han P., Wang X., Zhou T., Cheng J., Wang C., Sun F., Zhao X. (2022). Inhibition of ferroptosis attenuates oligospermia in male Nrf2 knockout mice. Free Radic. Biol. Med..

[82] Wang Z.-Y., Liu Y.-Y., Liu G.-H., Lu H.-B., Mao C.-Y. (2018). L-Carnitine and heart disease. Life Sci..

[83] Ferreira G.C., McKenna M.C. (2017). L-Carnitine and Acetyl-l-carnitine Roles and Neuroprotection in Developing Brain. Neurochem. Res..

[84] Fattorossi A., Biselli R., Casciaro A., Tzantzoglou S., de Simone C. (1993). Regulation of normal human polyrnorphonuclear leucocytes by carnitine. Mediat. Inflamm..

[85] Matalliotakis I., Koumantaki Y., Evageliou A., Matalliotakis G., Goumenou A., Koumantakis E. (2000). L-Carnitine levels in the seminal plasma of fertile and infertile men: Correlation with sperm quality. Int. J. Fertil. Women’s Med..

[86] Vicari E., Calogero A. (2001). Effects of treatment with carnitines in infertile patients with prostato-vesiculo-epididymitis. Hum. Reprod..

[87] Casillas E.R. (1972). The distribution of carnitine in male reproductive tissues and its effect on palmitate oxidation by spermatozoal particles. Biochim. Biophys. Acta (BBA)—Lipids Lipid Metab..

[88] Casillas E.R., Chaipayungpan S. (1979). The distribution of carnitine and acetylcarnitine in the rabbit epididymis and the carnitine content of rabbit spermatozoa during maturation. Reproduction.

[89] Hinton B.T., Brooks D.E., Dott H.M., Setchell B.P. (1981). Effects of carnitine and some related compounds on the motility of rat spermatozoa from the caput epididymidis. J. Reprod. Fertil..

[90] Palmero S., Bottazzi C., Costa M., Leone M., Fugassa E. (2000). Metabolic effects of L-Carnitine on prepubertal rat Sertoli cells. Horm. Metab. Res..

[91] Caviglia D., Scarabelli L., Palmero S. (2004). Effects of Carnitines on Rat Sertoli Cell Protein Metabolism. Horm. Metab. Res..

[92] Kobayashi D., Goto A., Maeda T., Nezu J.-I., Tsuji A., Tamai I. (2005). OCTN2-mediated transport of carnitine in isolated Sertoli cells. Reproduction.

[93] Khushboo M., Murthy M.K., Devi M.S., Sanjeev S., Ibrahim K.S., Kumar N.S., Roy V.K., Gurusubramanian G. (2018). Testicular toxicity and sperm quality following copper exposure in Wistar albino rats: Ameliorative potentials of L-Carnitine. Environ. Sci. Pollut. Res. Int..

[94] Karam K.M., Alebady A.S., Al-Nailey K.G.C., Al-Delemi D.H.J. (2022). L-Carnitine effect on induced hyperlipidemia on premature rats: Fertility profile. J. Med. Life.

[95] Abdel-Emam R.A., Ahmed E.A. (2021). Ameliorative effect of L-Carnitine on chronic lead-induced reproductive toxicity in male rats. Vet. Med. Sci..

[96] Longobardi V., Salzano A., Campanile G., Marrone R., Palumbo F., Vitiello M., Zullo G., Gasparrini B. (2017). Carnitine supplementation decreases capacitation-like changes of frozen-thawed buffalo spermatozoa. Theriogenology.

[97] Fu L.-L., Zhang L.-Y., An Q., Zhou F., Tong Y., Guo Y., Lu W.-H., Liang X.-W., Chang B., Gu Y.-Q. (2018). L-Carnitine protects the motion parameters and mitochondrial function of human sperm in cryopreservation. Zhonghua Nan Ke Xue = Natl. J. Androl..

[98] Abd-Elrazek A.M., Ahmed-Farid O.A.H. (2017). Protective effect of L-Carnitine and L-Arginine against busulfan-induced oligospermia in adult rat. Andrologia.

[99] Khademi A., Alleyassin A., Safdarian L., Hamed E.A., Rabiee E., Haghaninezhad H. (2005). The effects of L-Carnitine on sperm parameters in smoker and non-smoker patients with idiopathic sperm abnormalities. J. Assist. Reprod. Genet..

[100] Sun L.-L., Wan X.-X., Zhang Y., Zhang Y.-H., Zhao W.-J., Wang D., Wang J.-G., Xie J.-L., Ma H.-G. (2018). L-Carnitine improves sperm acrosin activity in male infertility patients. Zhonghua Nan Ke Xue = Natl. J. Androl..

[101] Nezhad N.C., Vahabzadeh Z., Allahveisie A., Rahmani K., Raoofi A., Rezaie M.J., Rezaei M., Partovyan M. (2021). The Effect of L-Carnitine and Coenzyme Q10 on the Sperm Motility, DNA Fragmentation, Chromatin Structure and Oxygen Free Radicals During, before and after Freezing in Oligospermia Men. Urol. J..

[102] Busetto G.M., Agarwal A., Virmani A., Antonini G., Ragonesi G., Del Giudice F., Micic S., Gentile V., De Berardinis E. (2018). Effect of metabolic and antioxidant supplementation on sperm parameters in oligo-astheno-teratozoospermia, with and without varicocele: A double-blind placebo-controlled study. Andrologia.

[103] Rebouche C.J., Shils M.E., Olson J.A., Shike M., Ross A.C. (1999). Carnitine. Modern Nutrition in Health and Disease.

[104] Alesci S., Manoli I., Costello R., Coates P., Gold P.W., Chrousos G.P., Blackman M.R. (2004). Carnitine: Lessons from one hundred years of research. Ann. N. Y. Acad. Sci..

[105] Diao R., Gan H., Tian F., Cai X., Zhen W., Song X., Duan Y. (2019). In vitro antioxidation effect of Quercetin on sperm function from the infertile patients with leukocytospermia. Am. J. Reprod. Immunol..

[106] Pasquariello R., Verdile N., Brevini T.A.L., Gandolfi F., Boiti C., Zerani M., Maranesi M. (2020). The Role of Resveratrol in Mammalian Reproduction. Molecules.

[107] Micic S., Lalic N., Djordjevic D., Bojanic N., Bogavac-Stanojevic N., Busetto G.M., Virmani A., Agarwal A. (2019). Double-blind, randomised, placebo-controlled trial on the effect of L-Carnitine and L-Acetylcarnitine on sperm parameters in men with idiopathic oligoasthenozoospermia. Andrologia.

